# Chaotic Attractors with Fractional Conformable Derivatives in the Liouville–Caputo Sense and Its Dynamical Behaviors

**DOI:** 10.3390/e20050384

**Published:** 2018-05-20

**Authors:** Jesús Emmanuel Solís Pérez, José Francisco Gómez-Aguilar, Dumitru Baleanu, Fairouz Tchier

**Affiliations:** 1 Tecnológico Nacional de México/CENIDET, Interior Internado Palmira S/N, Col. Palmira, C.P. 62490 Cuernavaca, Mexico; 2 CONACyT-Tecnológico Nacional de México/CENIDET, Interior Internado Palmira S/N, Col. Palmira, C.P. 62490 Cuernavaca, Mexico; 3 Department of Mathematics, Faculty of Art and Sciences, Cankaya University, 06530 Ankara, Turkey; 4 Institute of Space Sciences, P.O. Box, MG-23, R 76900 Magurele-Bucharest, Romania; 5 Department of Mathematics, King Saud University, P.O. BOX 2454, Riyadh 11451, Saudi Arabia

**Keywords:** fractional calculus, fractional conformable derivative, fractional *β*-conformable derivative, chaos, Adams–Moulton scheme

## Abstract

This paper deals with a numerical simulation of fractional conformable attractors of type Rabinovich–Fabrikant, Thomas’ cyclically symmetric attractor and Newton–Leipnik. Fractional conformable and β-conformable derivatives of Liouville–Caputo type are considered to solve the proposed systems. A numerical method based on the Adams–Moulton algorithm is employed to approximate the numerical simulations of the fractional-order conformable attractors. The results of the new type of fractional conformable and β-conformable attractors are provided to illustrate the effectiveness of the proposed method.

## 1. Introduction

Fractional derivatives, which are a generalization of classical derivatives have been extensively used in describing and solving integral equations, ordinary and partial differential equations in applied sciences such as fluid mechanics, diffusive transport, electrical networks, electrodynamics, nonlinear control theory, signal processing, nonlinear biological systems, astrophysics, among others [1,2,3,4,5,6,7,8,9,10,11,12,13,14,15,16,17,18,19].

Several definitions exist regarding the fractional derivatives, and some fundamental definitions are Coimbra, Riesz, Riemann–Liouville, Hadamard, Weyl, Grünwald–Letnikov, Marchaud, Liouville–Caputo, Caputo–Fabrizio, Atangana–Baleanu [20,21,22]. Based on the concept of local derivative with fractional components, Khalil presented the “conformable derivative” in [23]. This derivative allows for many extensions of some classical theorems in calculus (i.e., the product rule, quotient rule, Rolle’s theorem, chain rule, mean value theorem and composition rule). Atangana in [24] proposed a modified version of the conformable derivative proposed by Khalil, and this conformable derivative is called the β-derivative. This derivative depends on the interval on which the function is being differentiated. The conformable derivatives may not be seen as fractional derivative but can be considered to be a natural extension of the conventional derivative. Some interesting works involving these conformable derivatives have been reported in [25,26,27,28,29,30,31,32,33,34,35].

Recently, in [36], new fractional integration and differentiation operators in Riemann–Liouville, Hadamard and Liouville–Caputo sense were obtained iterating conformable integrals. These fractional conformable operators have properties similar to the classical calculus. An important advantage of these operators is that they depend on two fractional parameters naturally, which allows better detection of the memory of the physical systems. Fundamental properties of these fractional conformable derivatives and integrals are given in [36].

In this work, we propose a numerical algorithm based on the Adams–Moulton scheme together with the Runge–Kutta method to get a numerical solution and chaotic behaviors of the fractional conformable and β-conformable Rabinovich–Fabrikant, Thomas’ cyclically symmetric attractor and Newton–Leipnik attractor in the Liouville–Caputo sense. The paper is organized as follows: in Section 2, fractional derivatives and mathematical preliminaries are given. In Section 3, we apply the numerical method proposed to simulate the Rabinovich–Fabrikant attractor, Thomas’ cyclically symmetric attractor and Newton–Leipnik chaotic attractor. Finally, conclusions are given in Section 4.

## 2. Mathematical Preliminaries

Let us recall known definitions of fractional derivatives.

**Definition** **1.** *The Riemann–Liouville operator (RL) is the derivative of the convolution of a given function and a power-law kernel. The RL fractional derivative with order (α>0) is defined as follows [20]:*

(1) $${}_{a}^{RL}D_{t}^{\alpha }f\left(t\right)=\frac{1}{\Gamma (n-\alpha )}\frac{d^{n}}{dt^{n}}\int_{a}^{t}f\left(\theta \right){\left(t-\theta \right)}^{n-\alpha -1}d\theta ,\;n-1<\alpha <n.$$

**Definition** **2.** *The Liouville–Caputo operator (C) is the convolution of the local derivative of a given function with power-law function. The Liouville–Caputo fractional derivative with order (α>0) is defined as follows [20]:*

(2) $${}_{a}^{C}D_{t}^{\alpha }f\left(t\right)=\frac{1}{\Gamma (n-\alpha )}\int_{a}^{t}\frac{d^{n}}{d\theta^{n}}f\left(\theta \right){\left(t-\theta \right)}^{n-\alpha -1}d\theta ,\;n-1<\alpha <n.$$

**Definition** **3.** *Let f:(0,∞)⟶ℜ, then, the conformable derivative of f(t) with order (α>0) is given by [23]*

(3) $${}_{a}^{}D_{t}^{\alpha }f\left(t\right)=\lim_{\epsilon \to 0}\frac{f\left(t+\epsilon t^{1-\alpha }\right)-f\left(t\right)}{\epsilon },$$

*for all t>0, α∈(0,1). If f(t) is α-differentiable in some (0,a), a>0, and $\lim_{t\to 0^{+}}f^{\alpha }\left(t\right)$ exist, then we define $f^{\alpha }\left(0\right)=\lim_{t\to 0^{+}}f^{\alpha }\left(t\right)$.*

We shall present some properties of this new derivative:

1. $D_{\alpha }\left(af+bg\right)=aD_{\alpha }\left(f\right)+bD_{\alpha }\left(g\right)$, for all a,b∈ℜ.
2. $D_{\alpha }\left(t^{p}\right)=pt^{p-\alpha }$, for all p.
3. $D_{\alpha }\left(\Xi \right)=0$, if Ξ is a constant.
4. $D_{\alpha }\left(fg\right)=fD_{\alpha }\left(g\right)+gD_{\alpha }\left(f\right)$.
5. $D_{\alpha }\left(\frac{f}{g}\right)=\frac{gD_{\alpha }\left(f\right)-fD_{\alpha }\left(g\right)}{g^{2}}$.

**Definition** **4.** *The β-derivative is another type of conformable derivative. Let $f:[-\frac{a}{\Gamma (\alpha )},\infty )⟶R$, where f(t) and α are differentiable. Then, the conformable derivative of type β is given by [24]*

(4) $$\begin{array}{c} {}_{a}^{A}D_{t}^{\alpha }f\left(t\right)=\lim_{\epsilon \to 0}\frac{f\left(t+\epsilon {\left(t+\frac{1}{\Gamma (\alpha )}\right)}^{1-\alpha }\right)-f\left(t\right)}{\epsilon }, \end{array}$$

*where ${\left(t+\frac{1}{\Gamma (\alpha )}\right)}^{1-\alpha }$ describes the inhomogeneous time scale.*

**Definition** **5.** *Let* Re*(β)≥0, n=[Re(β)]+1, $f\in C_{\alpha ,a}^{n}\left([a,b]\right)$, $\left(f\in C_{\alpha ,b}^{n}\left([a,b]\right)\right)$. Then, the fractional conformable derivative in the Liouville–Caputo sense is given by [36]*

(5) $$\begin{array}{cc} {}_{\;\;a}^{c\;\beta }D_{t}^{\alpha }f\left(t\right) & =\frac{1}{\Gamma (n-\beta )}\int_{a}^{t}{\left(\frac{{\left(t-a\right)}^{\alpha }-{\left(x-a\right)}^{\alpha }}{\alpha }\right)}^{n-\beta -1}\frac{{}_{a}^{n}D_{x}^{\alpha }f\left(x\right)}{{\left(x-a\right)}^{1-\alpha }}\;dx,\\ & {=}_{\;\;\;\;\;a}^{c\;n-\beta }I_{t}^{\alpha }\left({\;}_{\;\;a}^{c\;n}D_{t}^{\alpha }f\left(t\right)\right). \end{array}$$

**Definition** **6.** *Let* Re*(β)≥0, n=[Re(β)]+1, $f\in C_{\alpha ,a}^{n}\left([a,b]\right)$, $\left(f\in C_{\alpha ,b}^{n}\left([a,b]\right)\right)$. Then, the fractional conformable derivative in the Riemann–Liouville sense is given by [36]*

(6) $$\begin{array}{cc} {}_{\;\;\;\;\;a}^{RL\;\beta }D_{t}^{\alpha }f\left(t\right) & =\frac{{}_{a}^{n}D_{t}^{\alpha }}{\Gamma (n-\beta )}\int_{a}^{t}{\left(\frac{{\left(t-a\right)}^{\alpha }-{\left(x-a\right)}^{\alpha }}{\alpha }\right)}^{n-\beta -1}\frac{f(x)}{{\left(x-a\right)}^{1-\alpha }}\;dx,\\ & {=}_{\;\;\;\;a}^{RL\;n}D_{t}^{\alpha }\left({}_{\;\;\;\;\;\;\;a}^{RL\;n-\beta }I_{t}f\left(t\right)\right). \end{array}$$

**Definition** **7.** *Let* Re*(β)≥0, n=[Re(β)]+1, $f\in C_{\alpha ,a}^{n}\left([a,b]\right)$, $\left(f\in C_{\alpha ,b}^{n}\left([a,b]\right)\right)$. Then, the fractional conformable derivative of β-type in the Liouville–Caputo sense (AC) is given by [37]*

(7) $$\begin{array}{cc} {}_{\;\;\;\;\;a}^{AC\;\beta }D_{t}^{\alpha }f\left(t\right) & =\frac{1}{\Gamma (n-\beta )}\int_{-\frac{a}{\Gamma (\alpha )}}^{t}{\left(\frac{{\left(t+\frac{a}{\Gamma (\alpha )}\right)}^{\alpha }-{\left(x+\frac{a}{\Gamma (\alpha )}\right)}^{\alpha }}{\alpha }\right)}^{n-\beta -1}\frac{{}_{\;\;\;a}^{A\;n}D_{x}^{\alpha }f\left(x\right)}{{\left(x+\frac{a}{\Gamma (\alpha )}\right)}^{1-\alpha }}\;dx,\\ & {=}_{\;\;\;\;\;\;a}^{A\;n-\beta }I_{t}^{\alpha }\left({\;}_{\;\;\;a}^{A\;n}D_{t}^{\alpha }f\left(t\right)\right). \end{array}$$

**Definition** **8.** *Let* Re*(β)≥0, n=[Re(β)]+1, $f\in C_{\alpha ,a}^{n}\left([a,b]\right)$, $\left(f\in C_{\alpha ,b}^{n}\left([a,b]\right)\right)$. Then, the fractional conformable derivative of β-type in the Riemann–Liouville sense (AR) is given by [37]*

(8) $$\begin{array}{cc} {}_{\;\;\;\;\;a}^{AR\;\beta }D_{t}^{\alpha }f\left(t\right) & =\frac{{}_{\;\;\;a}^{A\;n}D_{t}^{\alpha }}{\Gamma (n-\beta )}\int_{-\frac{a}{\Gamma (\alpha )}}^{t}{\left(\frac{{\left(t+\frac{a}{\Gamma (\alpha )}\right)}^{\alpha }-{\left(x+\frac{a}{\Gamma (\alpha )}\right)}^{\alpha }}{\alpha }\right)}^{n-\beta -1}\frac{f(x)}{{\left(x+\frac{a}{\Gamma (\alpha )}\right)}^{1-\alpha }}\;dx,\\ & {=}_{\;\;\;a}^{A\;n}D_{t}^{\alpha }\left({}_{\;\;\;\;\;\;a}^{A\;n-\beta }I_{t}f\left(t\right)\right). \end{array}$$

## 3. Adams–Moulton Scheme for Fractional Conformable Derivatives

Fractional Adams’ method [38,39,40] is a numerical algorithm for solving nonlinear fractional differential equation of the form

(9) $$D_{*}^{\alpha }y\left(t\right)=f\left(t,y\left(t\right)\right),\;\alpha >0,\;0\leq t\leq T,$$

with initial conditions

(10) $$y^{\left(k\right)}\left(0\right)=y_{0}^{\left(k\right)},$$

where k=0,1,2,3,⋯,⌈α⌉−1 and $D_{*}^{\alpha }$ denotes the operator of fractional derivative in the Liouville–Caputo sense. In addition, Equation (9) is equivalent to the Volterra integral equation of the second kind

(11) $$y\left(t\right)=\sum_{k=0}^{n-1}y_{0}^{\left(k\right)}\frac{t^{k}}{k!}+\frac{1}{\Gamma \left(\alpha \right)}\int_{0}^{t}{\left(t-s\right)}^{\alpha -1}f\left(s,y(s)\right)\;ds,\;n-1<\alpha \leq n.$$

To get a numerical solution for Equations (5) and (7), we propose the predictor scheme [41,42,43] used to discretize the Equation (11) and let us consider $h=\frac{T}{N}$, $t_{j}=jh$, j=0,1,2,3,⋯,N with *N* steps in an interval of [0,T], where $D_{*}^{\alpha }$ is the conformable derivative operator of Khalil’s or Atangana’s type.

Now, the new Adams–Moulton method [38,39,40] for the fractional conformable derivative in the Liouville–Caputo sense (FCAMM) is given as follows:

(12) $$x_{h}^{p}\left(t_{n+1}\right)=x_{0}+\frac{1}{\Gamma \left(\beta \right)}\sum_{j=0}^{n}b_{j,n+1}D_{*}^{\alpha }f\left(t_{j},x_{h}\left(t_{j}\right)\right),\begin{array}{c} \;0<\alpha <1,\\ \;0<\beta <1, \end{array}$$

where α denotes the order of the conformable derivative and β the fractional order of the Liouville–Caputo fractional derivative

(13) $$b_{j,n+1}=\frac{h^{\beta }}{\beta }\left[{\left(n+1-j\right)}^{\beta }-{\left(n-j\right)}^{\beta }\right],\;0\leq j\leq n.$$

In this work, we use the conformable derivative proposed by Khalil in Equation (3)

(14) $$D_{*}^{\alpha }f\left(t_{j},x_{h}\left(t_{j}\right)\right):=\left(t^{1-\alpha }\right)\frac{d}{dt}f\left(t_{j},x_{h}\left(t_{j}\right)\right),$$

and the β-conformable proposed by Atangana in Equation (4)

(15) $${}^{A}D_{*}^{\alpha }f\left(t_{j},x_{h}\left(t_{j}\right)\right):=\left.{\left(t+\frac{1}{\Gamma (\alpha )}\right)}^{1-\alpha }\right.\frac{d}{dt}f\left(t_{j},x_{h}\left(t_{j}\right)\right),$$

to solve numerically fractional differential equations involving a fractional conformable derivative and β-conformable derivative in the Liouville–Caputo sense.

## 4. Application and Numerical Examples

In this section, we consider some numerical experiments for different fractional-order values.

- *Rabinovich–Fabrikant attractor*. The model of Rabinovich–Fabrikant [44] was initially designed as a physical model describing the stochasticity arising from the modulation instability in a non-equilibrium dissipative medium. The Rabinovich–Fabrikant system is described by the following equations:
  (16) $$\begin{array}{c} \dot{x}=y\left(z-1+x^{2}\right)+ax,\\ \dot{y}=x\left(3z+1-x^{2}\right)+ay,\\ \dot{z}=-2z\left(b+xy\right), \end{array}$$
  where a,b>0. The system (16) is chaotic for some values of *a* and *b*, but for a<b, the system is dissipative.

**Figure 1:**
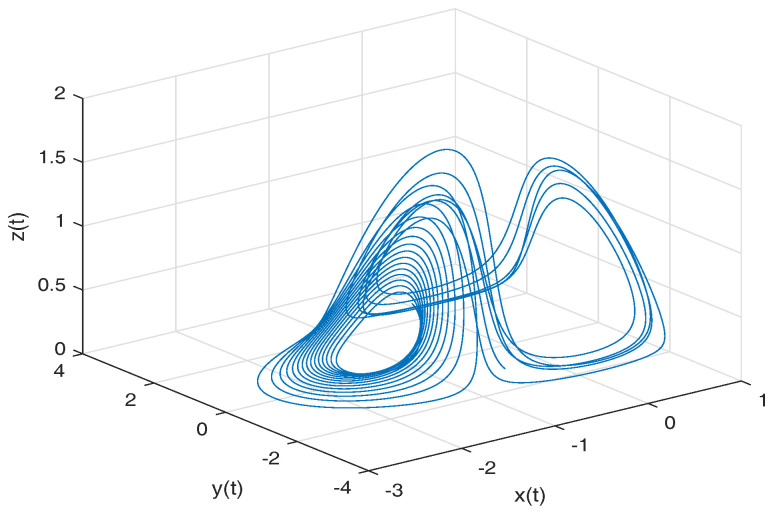
Numerical simulation for the Rabinovich–Fabrikant attractor (16) for a=0.10, b=0.14 with initial conditions x(0)=−1, y(0)=0, z(0)=0.5.

Figure 1 shows the numerical simulation of the Equation (16) for a=0.10, b=0.14, step size $h=5\times 10^{-3}$ and time simulation t=70[s], with initial conditions x(0)=−1, y(0)=0, z(0)=0.5.

If we replace the time derivative of the system (16) by the conformable derivative Equation (14) and β-conformable derivative Equation (15), then we get the following fractional conformable numerical schemes in the Liouville–Caputo sense.

- *Conformable sense:*
  (17) $$\begin{array}{c} {}_{\;\;0}^{c\;\beta }D_{t}^{\alpha }x=\frac{1}{\Phi }\left[y\left(z-1+x^{2}\right)+ax\right],\\ {}_{\;\;0}^{c\;\beta }D_{t}^{\alpha }y=\frac{1}{\Phi }\left[x\left(3z+1-x^{2}\right)+ay\right],\\ {}_{\;\;0}^{c\;\beta }D_{t}^{\alpha }z=\frac{1}{\Phi }\left[-2z\left(b+xy\right)\right]. \end{array}$$
  Using the numerical scheme (12), we represent the system (17) in the following form:
  (18) $$\begin{array}{c} x_{n+1}^{p}\left(t\right)=x_{0}\left(t\right)+\frac{1}{\Gamma \left(\beta \right)}\sum_{j=0}^{n}b_{1,j,n+1}f_{1}\left(x_{n},y_{n},z_{n},t_{n}\right),\\ y_{n+1}^{p}\left(t\right)=y_{0}\left(t\right)+\frac{1}{\Gamma \left(\beta \right)}\sum_{j=0}^{n}b_{2,j,n+1}f_{2}\left(x_{n},y_{n},z_{n},t_{n}\right),\\ z_{n+1}^{p}\left(t\right)=z_{0}\left(t\right)+\frac{1}{\Gamma \left(\beta \right)}\sum_{j=0}^{n}b_{3,j,n+1}f_{3}\left(x_{n},y_{n},z_{n},t_{n}\right), \end{array}$$
  where
  (19) $$\begin{array}{c} f_{1}\left(x_{n},y_{n},z_{n},t_{n}\right):=\frac{1}{\Phi }\left[y\left(z-1+x^{2}\right)+ax\right],\\ f_{2}\left(x_{n},y_{n},z_{n},t_{n}\right):=\frac{1}{\Phi }\left[x\left(3z+1-x^{2}\right)+ay\right],\\ f_{3}\left(x_{n},y_{n},z_{n},t_{n}\right):=\frac{1}{\Phi }\left[-2z\left(b+xy\right)\right], \end{array}$$
  and $\Phi :=t^{1-\alpha }$.
- *β-conformable sense:*
  (20) $$\begin{array}{c} {}_{\;\;\;\;\;0}^{AC\;\beta }D_{t}^{\alpha }x=\frac{1}{\psi }\left[y\left(z-1+x^{2}\right)+ax\right],\\ {}_{\;\;\;\;\;0}^{AC\;\beta }D_{t}^{\alpha }y=\frac{1}{\psi }\left[x\left(3z+1-x^{2}\right)+ay\right],\\ {}_{\;\;\;\;\;0}^{AC\;\beta }D_{t}^{\alpha }z=\frac{1}{\psi }\left[-2z\left(b+xy\right)\right]. \end{array}$$
  Using the numerical scheme (12), we represent the system (20) in the following form:
  (21) $$\begin{array}{c} x_{n+1}^{p}\left(t\right)=x_{0}\left(t\right)+\frac{1}{\Gamma \left(\beta \right)}\sum_{j=0}^{n}b_{1,j,n+1}g_{1}\left(x_{n},y_{n},z_{n},t_{n}\right),\\ y_{n+1}^{p}\left(t\right)=y_{0}\left(t\right)+\frac{1}{\Gamma \left(\beta \right)}\sum_{j=0}^{n}b_{2,j,n+1}g_{2}\left(x_{n},y_{n},z_{n},t_{n}\right),\\ z_{n+1}^{p}\left(t\right)=z_{0}\left(t\right)+\frac{1}{\Gamma \left(\beta \right)}\sum_{j=0}^{n}b_{3,j,n+1}g_{3}\left(x_{n},y_{n},z_{n},t_{n}\right), \end{array}$$
  where
  (22) $$\begin{array}{c} g_{1}\left(x_{n},y_{n},z_{n},t_{n}\right):=\frac{1}{\psi }\left[y\left(z-1+x^{2}\right)+ax\right],\\ g_{2}\left(x_{n},y_{n},z_{n},t_{n}\right):=\frac{1}{\psi }\left[x\left(3z+1-x^{2}\right)+ay\right],\\ g_{3}\left(x_{n},y_{n},z_{n},t_{n}\right):=\frac{1}{\psi }\left[-2z\left(b+xy\right)\right], \end{array}$$
  and $\psi :={\left(t+\frac{1}{\Gamma (\alpha )}\right)}^{1-\alpha }$.
- *Observation*. In the case when α→1, we obtain the numerical solution of the Rabinovich–Fabrikant attractor in the Liouville–Caputo sense.

**Figure 2:**
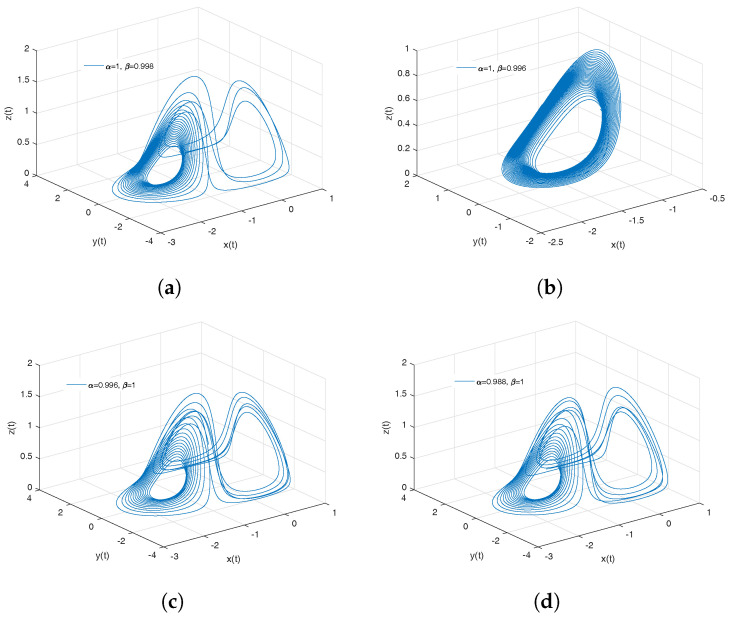
Numerical simulation for the scheme given by Equation (18) for a=0.10, b=0.14 with initial conditions x(0)=−1, y(0)=0, z(0)=0.5, for different particular cases of α and β. In (**a**), α=1, β=0.998. In (**b**), α=1, β=0.997. In (**c**), α=0.996, β=1. In (**d**), α=0.988, β=1, all values were arbitrarily chosen.

Figure 2a–d show numerical simulations from the Equation (18) for a=0.10, b=0.14, step size $h=5\times 10^{-3}$ and time simulation t=70[s], with initial conditions x(0)=−1, y(0)=0, z(0)=0.5, for different particular cases of α and β, arbitrarily chosen.

To develop the simulations shown in the Figure 2a,b, we consider α=1 and β≠1; in this case, both simulations show numerical solutions for the Rabinovich–Fabrikant attractor in the Liouville–Caputo sense. To develop the simulations shown in Figure 2c,d, we consider α≠1 and β=1; in this case, both simulations show numerical solutions for the conformable Rabinovich–Fabrikant attractor.

**Figure 3:**
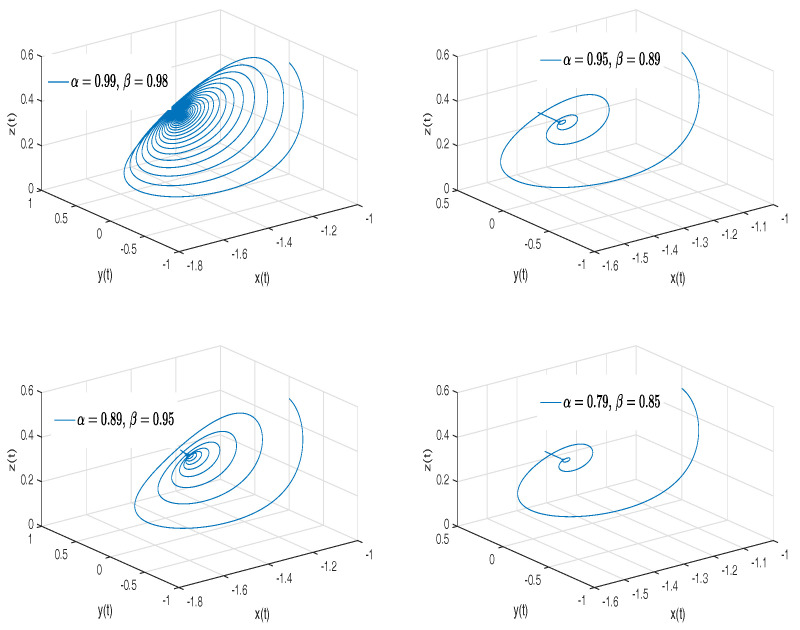
Numerical simulation for the Rabinovich–Fabrikant attractor (18) for a=0.10, b=0.14 with initial conditions x(0)=−1, y(0)=0, z(0)=0.5, for different particular cases of α and β, all values were arbitrarily chosen.

Figure 3 shows numerical simulations from the Equation (18) for a=0.10, b=0.14, step size $h=5\times 10^{-3}$ and time simulation t=70[s], with initial conditions x(0)=−1, y(0)=0, z(0)=0.5, for different particular cases of α and β, arbitrarily chosen.

**Figure 4:**
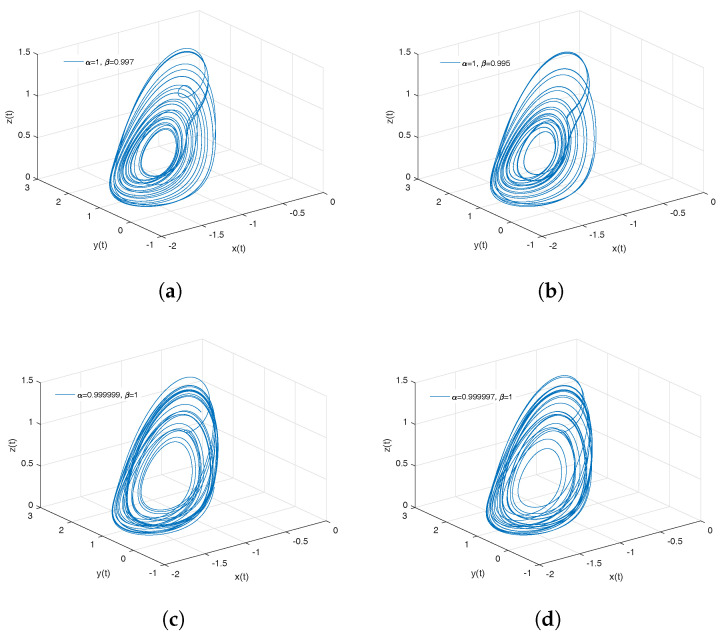
Numerical simulation for the scheme given by Equation (21) for a=0.10, b=0.14 with initial conditions x(0)=−1, y(0)=0, z(0)=0.5, for different particular cases of α and β. In (**a**) α=1, β=0.997. In (**b**) α=1, β=0.995. In (**c**) α=0.994, β=1. In (**d**) α=0.992, β=1, all values were arbitrarily chosen.

Figure 4a,b shows numerical simulations from the Equation (21) for a=0.10, b=0.14, step size $h=5\times 10^{-3}$ and time simulation t=70[s], with initial conditions x(0)=−1, y(0)=0, z(0)=0.5, for different particular cases of α and β, arbitrarily chosen.

To develop the simulations shown in Figure 4a,b, we consider α=1 and β≠1; in this case, both simulations show numerical solutions for the Rabinovich–Fabrikant attractor in the Liouville–Caputo sense. To develop the simulations shown in the Figure 4c,d, we consider α≠1 and β=1; in this case, both simulations show numerical solutions for the β-conformable Rabinovich–Fabrikant attractor.

**Figure 5:**
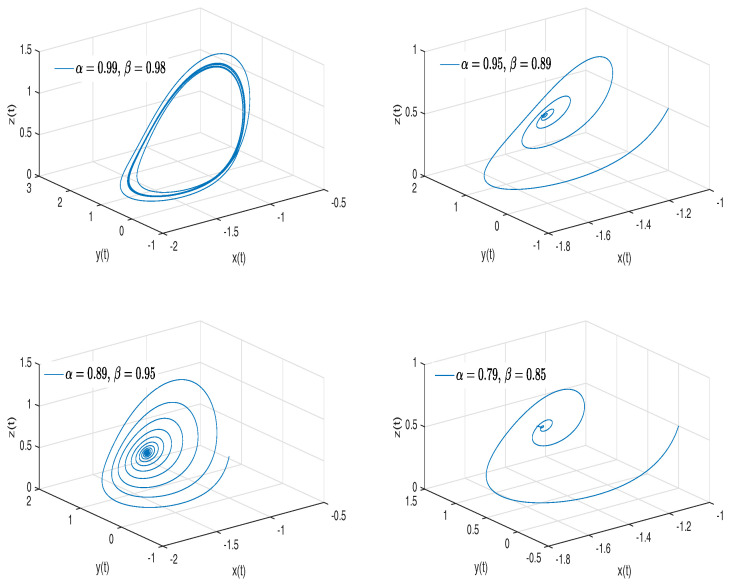
Numerical simulation for the Rabinovich–Fabrikant attractor (21) for a=0.10, b=0.14 with initial conditions x(0)=−1, y(0)=0, z(0)=0.5, for different particular cases of α and β, all values were arbitrarily chosen.

Figure 5 shows numerical simulations from the Equation (21) for a=0.10, b=0.14, step size $h=5\times 10^{-3}$ and time simulation t=70[s], with initial conditions x(0)=−1, y(0)=0, z(0)=0.5, for different particular cases of α and β, arbitrarily chosen.

Self-excited attractors can be visualized numerically, in which, after a transient process a trajectory, starting from a point of a neighborhood of unstable equilibrium, attracted to the attractor. According to the election of the orders of derivation, we have illustrated that the system may possess multiple topologically different chaotic attractors.

- *Thomas’ cyclically symmetric attractor.* Thomas in [45] proposed a mathematically three-dimensional cyclically symmetric attractor. This system is cyclically symmetric in the variables *x*, *y*, and *z* and considers a frictional damping *b*. The Thomas’ cyclically symmetric attractor is described by the following equations:
  (23) $$\begin{array}{c} \dot{x}=\sin\left(y\right)-bx,\\ \dot{y}=\sin\left(z\right)-by,\\ \dot{z}=\sin\left(x\right)-bz, \end{array}$$
  where *b* can be considered a frictional damping for a particle moving in a three-dimensional lattice [46]. This attractor is tuned by a single value in any dimension of range 2 to 3; it also has the quality of transition from a dissipative system to a conservative system.

**Figure 6:**
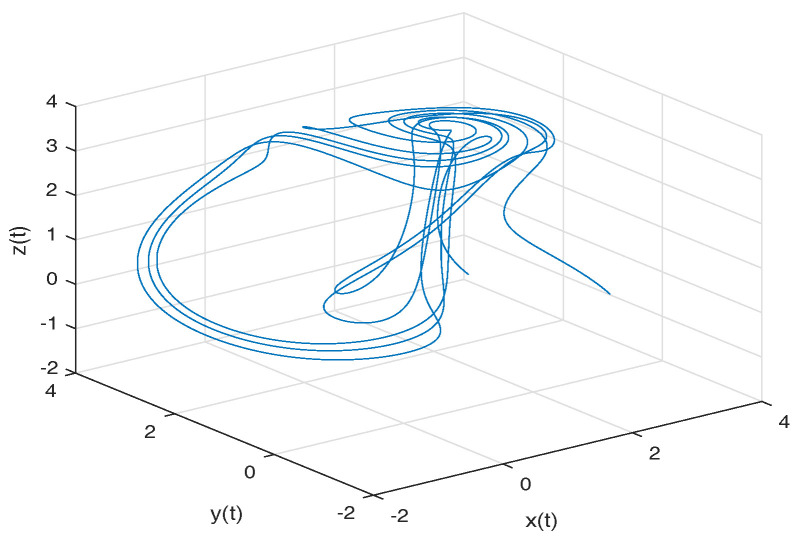
Numerical simulation for the Thomas’ cyclically symmetric attractor (23) for b=0.1998, step size $h=1\times 10^{-2}$, simulation time t=150[s] and initial conditions x(0)=1, y(0)=0z(0)=1.

Figure 6 shows the numerical simulation of the Equation (23) for b=0.1998, step size $h=1\times 10^{-2}$, simulation time t=150[s] and initial conditions x(0)=1, y(0)=0z(0)=1.

Applying the operator ${}_{\;\;0}^{c\;\beta }D_{t}^{\alpha }$ to Equation (23), we get

(24) $$\begin{array}{c} {}_{\;\;0}^{c\;\beta }D_{t}^{\alpha }x=\frac{1}{\Phi }\left[\sin\left(y\right)-bx\right],\\ {}_{\;\;0}^{c\;\beta }D_{t}^{\alpha }y=\frac{1}{\Phi }\left[\sin\left(z\right)-by\right],\\ {}_{\;\;0}^{c\;\beta }D_{t}^{\alpha }z=\frac{1}{\Phi }\left[\sin\left(x\right)-bz\right]. \end{array}$$

If we proceed in a similar way applying the operator ${}_{\;\;\;\;\;a}^{AC\;\beta }D_{t}^{\alpha }$, then we have

(25) $$\begin{array}{c} {}_{\;\;\;\;\;0}^{AC\;\beta }D_{t}^{\alpha }x=\frac{1}{\psi }\left[\sin\left(y\right)-bx\right],\\ {}_{\;\;\;\;\;0}^{AC\;\beta }D_{t}^{\alpha }y=\frac{1}{\psi }\left[\sin\left(z\right)-by\right],\\ {}_{\;\;\;\;\;0}^{AC\;\beta }D_{t}^{\alpha }z=\frac{1}{\psi }\left[\sin\left(x\right)-bz\right]. \end{array}$$

Applying FCAMM to Equations (24) and (25), we set the parameters of Thomas’ attractor as b=0.1998, step size $h=1\times 10^{-2}$, simulation time t=150[s] and initial conditions x(0)=1, y(0)=0z(0)=1, we get the numerical solution for the conformable systems (24) and (25).

- *Observation*. In the case when α→1, we obtain the numerical solution of the Thomas’ cyclically symmetric attractor in the Liouville–Caputo sense.

**Figure 7:**
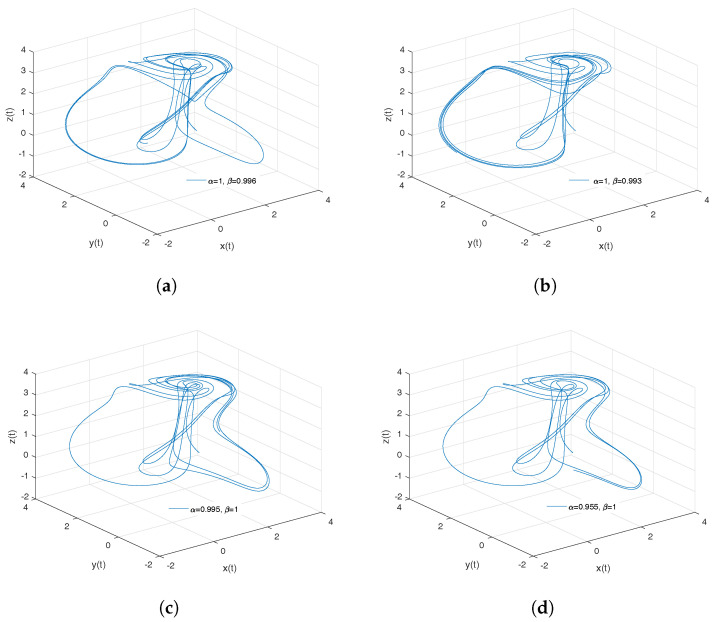
Numerical simulation for the scheme given by Equation (24) for b=0.1998, step size $h=1\times 10^{-2}$, simulation time t=150[s] and initial conditions x(0)=1, y(0)=0z(0)=1, for different particular cases of α and β. In (**a**), α=1, β=0.996. In (**b**), α=1, β=0.993. In (**c**), α=0.995, β=1. In (**d**), α=0.955, β=1, all values were arbitrarily chosen.

Figure 7a–d shows numerical simulations from the Equation (24) for b=0.1998, step size $h=1\times 10^{-2}$, simulation time t=150[s] and initial conditions x(0)=1, y(0)=0z(0)=1, for different particular cases of α and β, arbitrarily chosen.

To develop the simulations shown in the Figure 7a,b, we consider α=1 and β≠1, in this case, both simulations show numerical solutions for the Thomas’ cyclically symmetric attractor in the Liouville–Caputo sense. To develop the simulations shown in the Figure 7c,d, we consider α≠1 and β=1, in this case, both simulations show numerical solutions for the conformable Thomas’ cyclically symmetric attractor.

**Figure 8:**
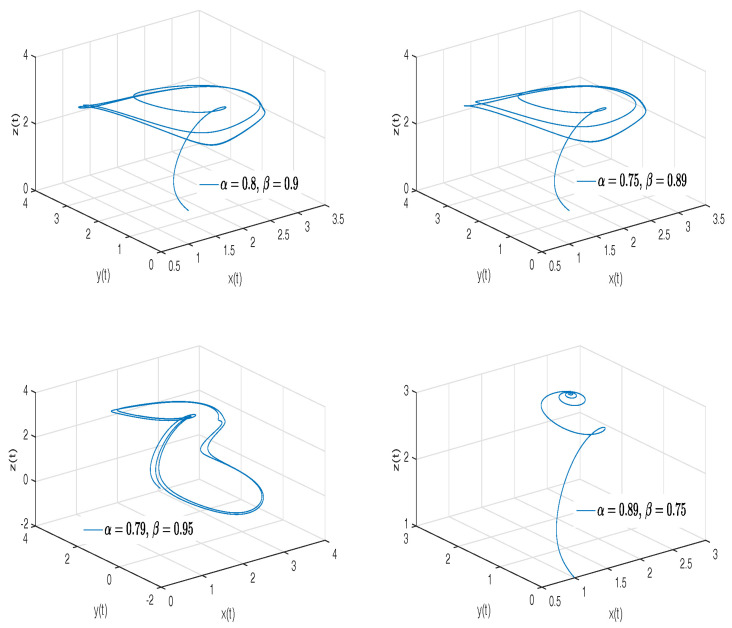
Numerical simulation for the Thomas’ cyclically symmetric attractor (24) for b=0.1998, step size $h=1\times 10^{-2}$, simulation time t=150[s] and initial conditions x(0)=1, y(0)=0z(0)=1, for different particular cases of α and β, all values were arbitrarily chosen.

Figure 8 shows numerical simulations from the Equation (24) for b=0.1998, step size $h=1\times 10^{-2}$, simulation time t=150[s] and initial conditions x(0)=1, y(0)=0z(0)=1, for different particular cases of α and β, arbitrarily chosen.

**Figure 9:**
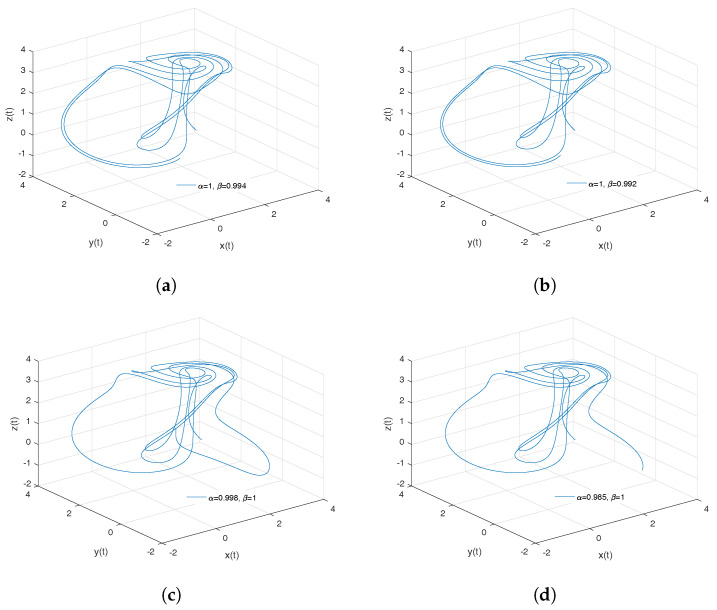
Numerical simulation for the scheme given by Equation (25) for b=0.1998, step size $h=1\times 10^{-2}$, simulation time t=150[s] and initial conditions x(0)=1, y(0)=0z(0)=1, for different particular cases of α and β. In (**a**), α=1, β=0.994. In (**b**), α=1, β=0.992. In (**c**), α=0.994, β=1. In (**d**), α=0.985, β=1, all values were arbitrarily chosen.

Figure 9a,b shows numerical simulations from the Equation (25) for b=0.1998, step size $h=1\times 10^{-2}$, simulation time t=150[s] and initial conditions x(0)=1, y(0)=0z(0)=1, for different particular cases of α and β, arbitrarily chosen.

To develop the simulations shown in the Figure 9a,b, we consider α=1 and β≠1, in this case, both simulations show numerical solutions for the Thomas’ cyclically symmetric attractor in the Liouville–Caputo sense. To develop the simulations shown in the Figure 9c,d, we consider α≠1 and β=1, in this case, both simulations show numerical solutions for the β-conformable Thomas’ cyclically symmetric attractor.

**Figure 10:**
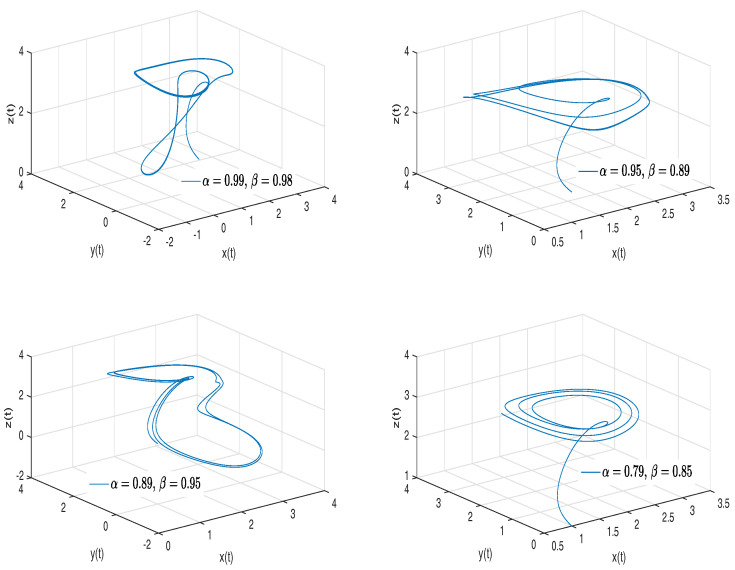
Numerical simulation for the Thomas’ cyclically symmetric attractor (25) for b=0.1998, step size $h=1\times 10^{-2}$, simulation time t=70[s] and initial conditions x(0)=−1, y(0)=0z(0)=0.5, for different particular cases of α and β, all values were arbitrarily chosen.

Figure 10 shows numerical simulations from the Equation (25) for a=0.10, b=0.14, step size $h=5\times 10^{-3}$, time simulation t=70[s], with initial conditions x(0)=−1, y(0)=0, z(0)=0.5, for different particular cases of α and β, arbitrarily chosen.

The Thomas’ cyclically symmetric attractor has a single parameter *b* that controls the damping and that is a natural bifurcation parameter for studying the route to chaos. For this example, the figures show that when α<1 and β<1, the numerical results presented to continual transition from a chaotic dissipative systems. This is because the different values of α and β modified the damping capacity of the systems. For example, when α and β are equal to 0.996, the damping capacity is bigger than when α and β are equal to 0.999.

- *Newton–Leipnik attractor.* The Newton–Leipnik system model was obtained by modifying Euler’s rigid body equations with the addition of a linear feedback in 1981. For this example, we consider a 3D system of fractional order nonlinear autonomous differential equations known as Newton–Leipnik attractor [47,48]:
  (26) $$\begin{array}{c} \dot{x}=-ax+y+cyz,\\ \dot{y}=-x-ay+dxz,\\ \dot{z}=bz-dxy, \end{array}$$
  where (a,c,d)∈R.

**Figure 11:**
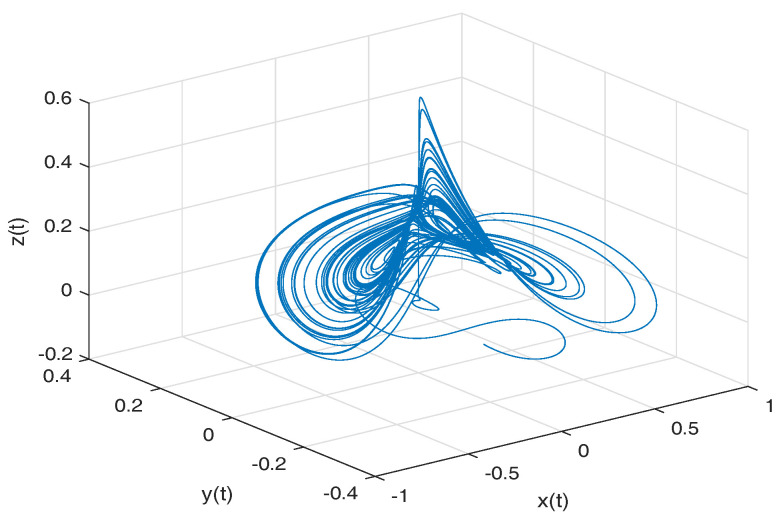
Numerical simulation for the Newton–Leipnik attractor (26) for a=0.4, b=0.175, c=10, d=5, step size $h=1\times 10^{-2}$, simulation time t=400[s] and initial conditions x(0)=0.349, y(0)=0, z(0)=−0.16.

Figure 11 shows the numerical simulation for the Equation (26) a=0.4, b=0.175, c=10, d=5, step size $h=1\times 10^{-2}$, simulation time t=400[s] and initial conditions x(0)=0.349, y(0)=0, z(0)=−0.16.

Replacing the time derivative in Equation (26) by the conformable operators Equations (14) and (15), we get the following fractional conformable numerical solutions in the Liouville–Caputo sense:

(27) $$\begin{array}{c} {}_{\;\;0}^{c\;\beta }D_{t}^{\alpha }x=-ax+y+cyz,\\ {}_{\;\;0}^{c\;\beta }D_{t}^{\alpha }y=-x-ay+dxz,\\ {}_{\;\;0}^{c\;\beta }D_{t}^{\alpha }z=bz-dxy, \end{array}$$

and, for β-conformable in the Liouville–Caputo sense, we have

(28) $$\begin{array}{c} {}_{\;\;\;\;\;0}^{AC\;\beta }D_{t}^{\alpha }x=-ax+y+cyz,\\ {}_{\;\;\;\;\;0}^{AC\;\beta }D_{t}^{\alpha }y=-x-ay+dxz,\\ {}_{\;\;\;\;\;0}^{AC\;\beta }D_{t}^{\alpha }z=bz-dxy. \end{array}$$

We assume that the systems represented in Equations (27) and (28) generate a chaotic attractor because we consider a=0.4, b=0.175, c=10, d=5 and initial conditions x(0)=0.349, y(0)=0, z(0)=−0.16. The above equations can be solved numerically using the predictor scheme given in Equation (12) considering a step size at $h=1\times 10^{-2}$ and simulation time t=400[s].

- *Observation*. In the case when α→1, we obtain the numerical solution of the Newton–Leipnik attractor in the Liouville–Caputo sense.

**Figure 12:**
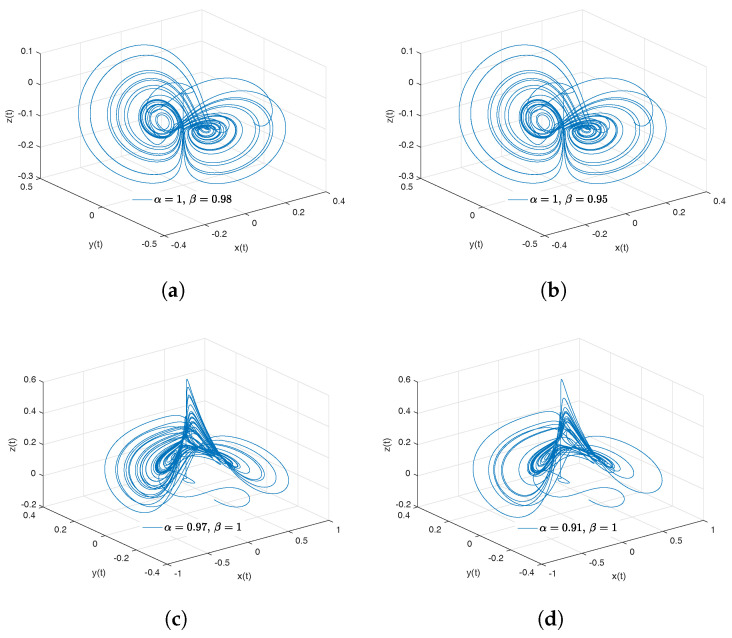
Numerical simulation for the scheme given by Equation (27) for a=0.4, b=0.175, c=10, d=5, step size $h=1\times 10^{-2}$, simulation time t=400[s] and initial conditions x(0)=0.349, y(0)=0, z(0)=−0.16, for different particular cases of α and β. In (**a**), α=1, β=0.98. In (**b**), α=1, β=0.95. In (**c**), α=0.97, β=1. In (**d**), α=0.91, β=1, all values were arbitrarily chosen.

Figure 12a–d shows numerical simulations from the Equation (27) for a=0.4, b=0.175, c=10, d=5, step size $h=1\times 10^{-2}$, simulation time t=400[s] and initial conditions x(0)=0.349, y(0)=0, z(0)=−0.16, for different particular cases of α and β, arbitrarily chosen.

To develop the simulations shown in the Figure 12a,b, we consider α=1 and β≠1, in this case, both simulations show numerical solutions for the Newton–Leipnik attractor in the Liouville–Caputo sense. To develop the simulations shown in the Figure 12c,d, we consider α≠1 and β=1, in this case, both simulations show numerical solutions for the conformable Newton–Leipnik attractor.

**Figure 13:**
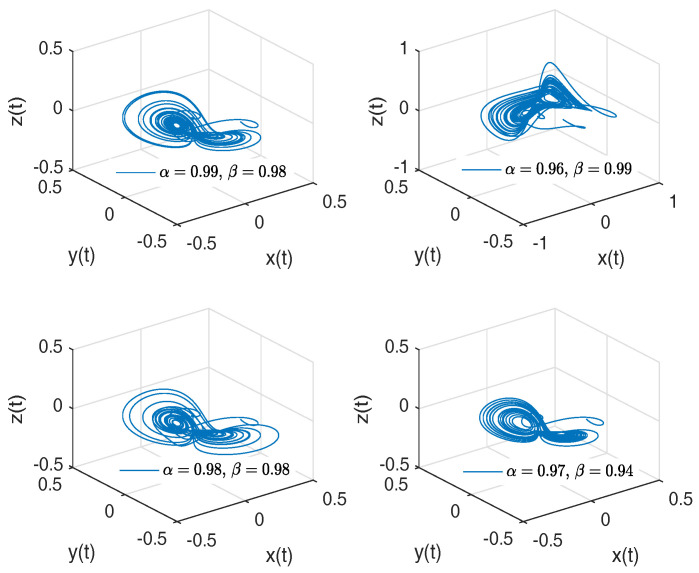
Numerical simulation for the Newton–Leipnik attractor (27) for a=0.4, b=0.175, c=10, d=5, step size $h=1\times 10^{-2}$, simulation time t=400[s] and initial conditions x(0)=0.349, y(0)=0, z(0)=−0.16, for different particular cases of α and β, all values were arbitrarily chosen.

Figure 13 shows numerical simulations from the Equation (27) for a=0.4, b=0.175, c=10, d=5, step size $h=1\times 10^{-3}$, simulation time t=400[s] and initial conditions x(0)=0.349, y(0)=0, z(0)=−0.16, for different particular cases of α and β, arbitrarily chosen.

**Figure 14:**
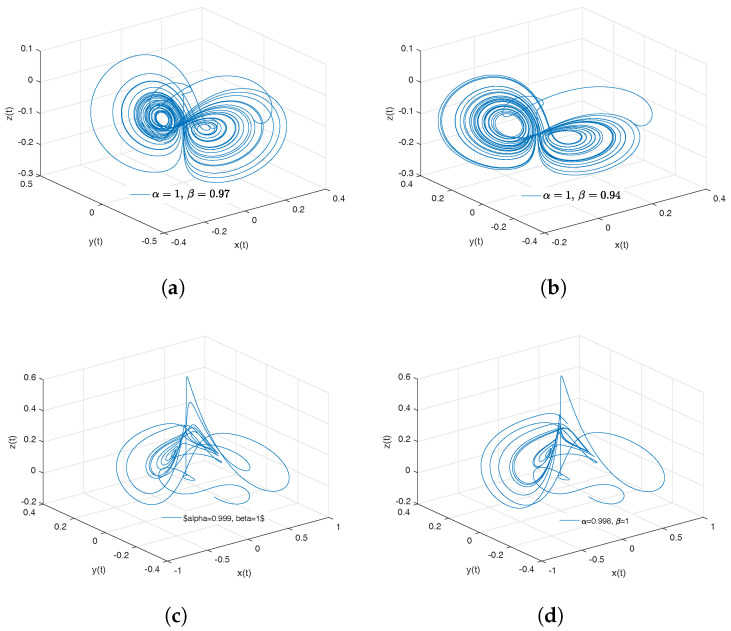
Numerical simulation for the scheme given by Equation (28) for a=0.4, b=0.175, c=10, d=5, step size $h=1\times 10^{-2}$, simulation time t=400[s] and initial conditions x(0)=0.349, y(0)=0, z(0)=−0.16, for different particular cases of α and β. In (**a**), α=1, β=0.97. In (**b**), α=1, β=0.94. In (**c**), α=0.999, β=1. In (**d**), α=0.9998, β=1, all values were arbitrarily chosen.

Figure 14a,b shows numerical simulations from the Equation (28) for a=0.4, b=0.175, c=10, d=5, step size $h=1\times 10^{-2}$, simulation time t=400[s] and initial conditions x(0)=0.349, y(0)=0, z(0)=−0.16, for different particular cases of α and β, arbitrarily chosen.

To develop the simulations shown in the Figure 14a,b, we consider α=1 and β≠1, in this case, both simulations show numerical solutions for the Newton–Leipnik attractor in the Liouville–Caputo sense. To develop the simulations shown in the Figure 14c,d, we consider α≠1 and β=1, in this case, both simulations show numerical solutions for the β-conformable Newton–Leipnik attractor.

**Figure 15:**
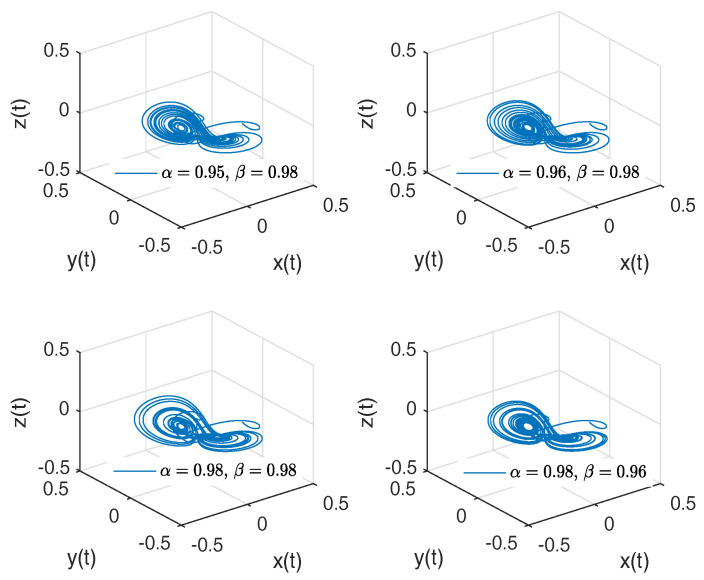
Numerical simulation for the Newton–Leipnik attractor (28) for a=0.4, b=0.175, c=10, d=5, step size $h=1\times 10^{-2}$, simulation time t=400[s] and initial conditions x(0)=0.349, y(0)=0, z(0)=−0.16, for different particular cases of α and β, all values were arbitrarily chosen.

Figure 15 shows numerical simulations from the Equation (28) for a=0.4, b=0.175, c=10, d=5, step size $h=5\times 10^{-2}$, time simulation t=400[s], with initial conditions x(0)=0.349, y(0)=0, z(0)=−0.16, for different particular cases of α and β, arbitrarily chosen.

The simulation results demonstrate that chaos indeed exists in the fractional-order system with order α and β less than 3. It was found that when 0.91<α,β<1, Newton–Leipnik attractor shows chaotic behavior. Furthermore, numerical simulations suggest that there exists both upper and lower attracting sets. This system display rich dynamic behaviors, such as periodic motions, chaotic motions, and transient chaos.

**Figure 16:**
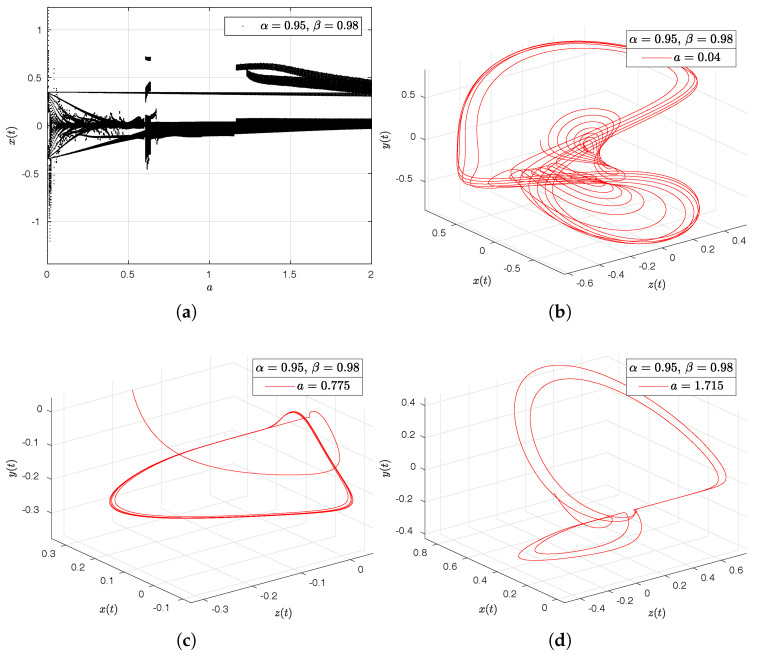
Bifurcation diagram of parameter *a* and portraits phase for the Newton–Leipnik system. In (**a**), bifurcation diagram of parameter *a*. In (**b**), portrait phase of the Newton–Leipnik system with α=0.95, β=0.98 and a=0.04. In (**c**), portrait phase of the Newton–Leipnik system with α=0.95, β=0.98 and a=0.775. In (**d**), portrait phase of the Newton–Leipnik system with α=0.95, β=0.98 and a=1.715, all values were arbitrarily chosen.

**Figure 17:**
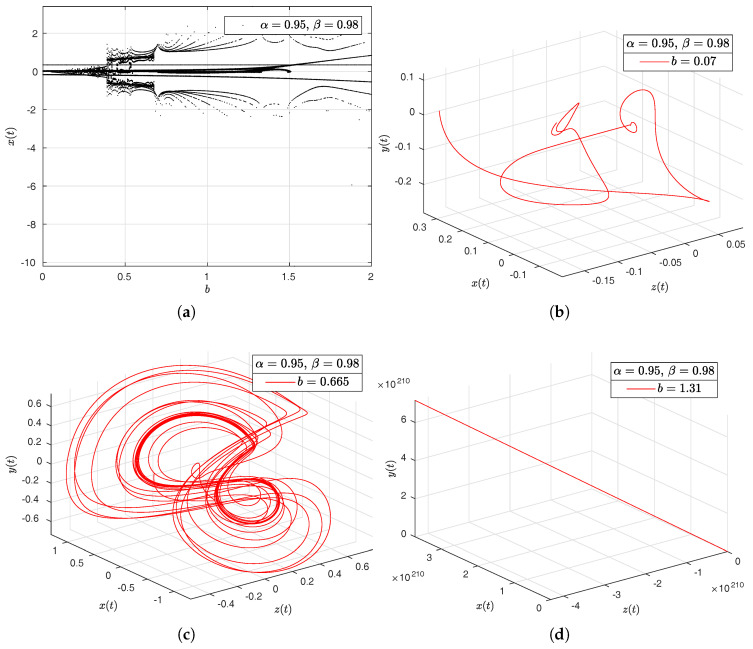
Bifurcation diagram of parameter *b* and portraits phase for the Newton–Leipnik system. In (**a**), bifurcation diagram of parameter *b*. In (**b**), portrait phase of the Newton–Leipnik system with α=0.95, β=0.98 and b=0.07. In (**c**), portrait phase of the Newton–Leipnik system with α=0.95, β=0.98 and b=0.665. In (**d**), portrait phase of the Newton–Leipnik system with α=0.95, β=0.98 and b=1.31, all values were arbitrarily chosen.

**Figure 18:**
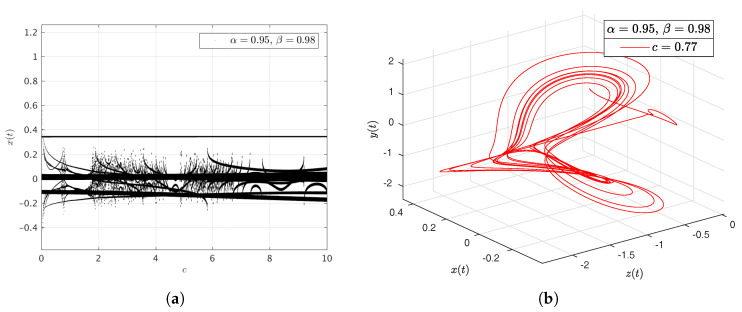

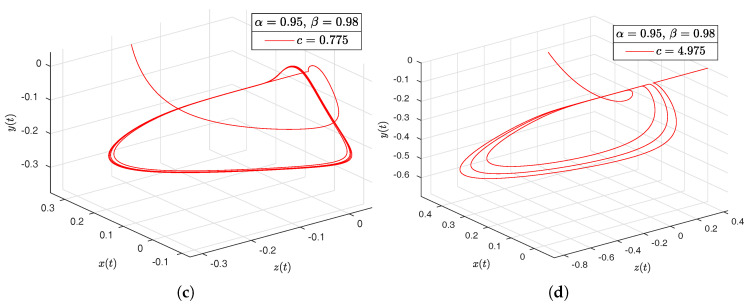
Bifurcation diagram of parameter *c* and portraits phase for the Newton–Leipnik system. In (**a**), bifurcation diagram of parameter *c*. In (**b**), portrait phase of the Newton–Leipnik system with α=0.95, β=0.98 and c=0.77. In (**c**), portrait phase of the Newton–Leipnik system with α=0.95, β=0.98 and c=0.775. In (**d**), portrait phase of the Newton–Leipnik system with α=0.95, β=0.98 and c=4.975, all values were arbitrarily chosen.

**Figure 19:**
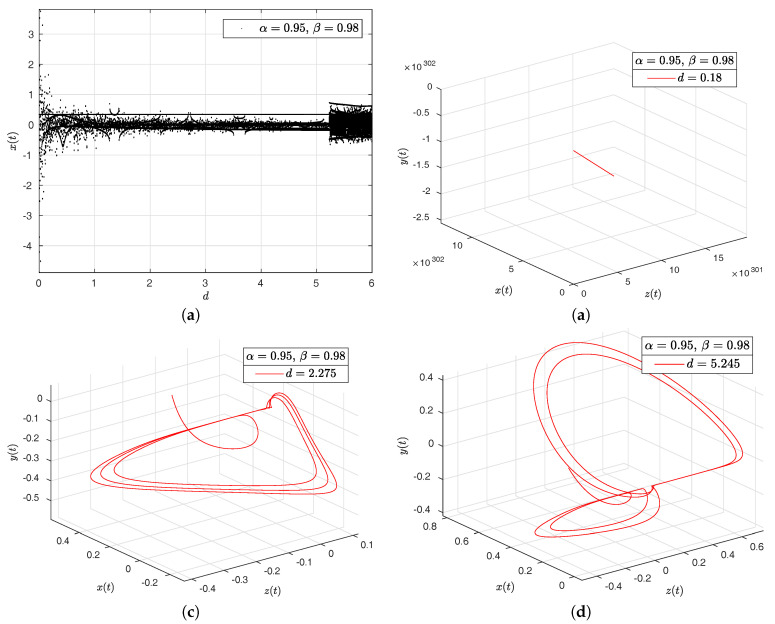
Bifurcation diagram of parameter *d* and portraits phase for the Newton–Leipnik system. In (**a**), bifurcation diagram of parameter *d*. In (**b**), portrait phase of the Newton–Leipnik system with α=0.95, β=0.98 and d=0.18. In (**c**), portrait phase of the Newton–Leipnik system with α=0.95, β=0.98 and b=2.275. In (**d**), portrait phase of the Newton–Leipnik system with α=0.95, β=0.98 and d=5.245, all values were arbitrarily chosen.

In the Figure 16a–d, Figure 17a–d, Figure 18a–d and Figure 19a–d we have computed the bifurcation diagrams with respect to parameters *a*, *b*, *c* and *d*.

The Euclidean distance is like an index of similarity between two points in the Euclidean space; hence, we represent the dynamic of the state *x* as a vector whose relation to the Euclidean space is natural and isomorphic. With this distance, we can perform a sensitivity analysis to the variation of initial conditions in the Newton–Leipnik system [49,50].

Assuming that $X(x_{1},x_{2},\cdots ,x_{n})$ is a point in an *n*-dimensional Euclidean space as a result of the dynamics of the Newton–Leipnik system with initial conditions: x(0)=0.349, y(0)=0 and z(0)=−0.16; parameters: a=0.4, b=0.175, c=10 and d=5; $X_{d}\;\left(x_{1d},x_{2d},\cdots ,x_{nd}\right)$ as an-other point in the Euclidean space whose dynamics depend on the initial condition $x_{0}\in \left[0.2,1\right]$ with $\Delta x_{0}=1\times 10^{-3}$, we can define the distance between the two dynamics *X* and $X_{d}$ as

(29) $$d\left(X,X_{d}\right):={\left(\sum_{i=1}^{n}{\left(x_{i}-x_{id}\right)}^{2}\right)}^{1/2}.$$

By using Equation (29), Euclidean space becomes a metric space where the following aphorisms can be satisfied

- If the distance between two points is larger than 0
  $$d\left(X,X_{d}\right)\geq 0,$$
  we assume that the dynamics of the system (27) are different; therefore, said system is susceptible to the change of initial conditions.
- The distance between two points is equal to 0, if and only if two points are overlapped
  $$\left(X,X_{d}\right)=0\;iff\;X=X_{d},$$
  wich means that the dynamics of the system are the same in that initial condition.

**Figure 20:**
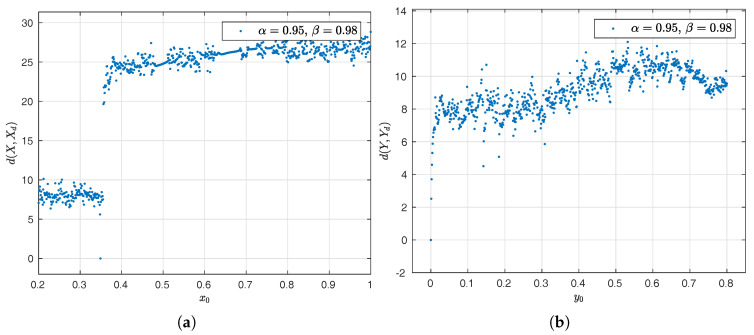

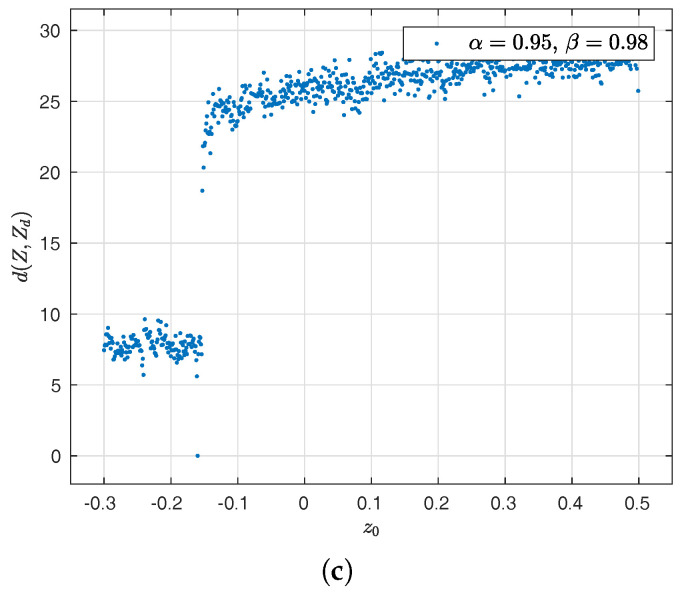
Visual projection distance of the different initial conditions along the time for the Newton–Leipnik system. In (**a**), initial condition $x_{0}$. In (**b**), initial condition $y_{0}$. In (**c**), initial condition $z_{0}$.

The spatial display is illustrated in the Figure 20a–c.

## 5. Conclusions

Within the framework of the fractional conformable differentiation, a modification of the Adams–Moulton method was suggested to solve fractional conformable differential equations, in particular chaotic systems of type Rabinovich–Fabrikant, Thomas’ cyclically symmetric attractor and Newton–Leipnik. The numerical scheme based in the Adams method permits solved numerically fractional conformable differential equations in the Liouville–Caputo sense. The modified numerical method is a mixture of the Adams–Moulton and the Runge–Kutta method. The method is accurate and efficient, direct, concise and converges quickly to the exact solution. At this point, to the best of the authors’ knowledge, there is no known or published numerical methods for solving fractional conformable differential equations in the Liouville–Caputo sense.

The dynamics of the Rabinovich–Fabrikant, Thomas’ cyclically symmetric attractor and Newton–Leipnik chaotic systems using fractional order conformable derivatives and β-conformable derivatives were studied numerically. We consider a novel fractional conformable and β-conformable derivatives of type Liouville–Caputo to investigate new types of chaotic behaviors. The novel fractional attractors depend naturally on two fractional parameters α and β; therefore, the systems studied display novel dynamic behaviors, periodic motions, chaotic motions, and transient chaos.

In the cases when α=1 and β≠1, we obtain chaotic motions described by the Liouville–Caputo fractional derivative. In the cases, when α≠1 and β=1, we obtain chaotic motions described by the conformable or β-conformable derivatives. Finally, in the case when α≠1 and β≠1, we obtain chaotic motions of type fractional conformable or fractional β-conformable in the Liouville–Caputo sense. We used some theoretical parameters to show the numerical simulations of fractional conformable and β-conformable attractors. We showed that, for certain values of parameters, the systems are chaotic and, for others, the systems tends to a stable periodic orbit. The systems considered produce rich dynamics that can serve as a prototype for chaos studies. These fractional chaotic motions based on the conformable and β-conformable derivatives in the Liouville–Caputo sense are showed for the first time in this work.

Our graphical representations explicitly reveal the complete reliability and efficiency of the presented method with a great potential in scientific applications. The new fractional conformable operators have become an important mathematical tool, motivated by the potential use for physicists and engineers working in various areas of the natural sciences. The chaos control of chaotic systems, the theoretical analysis of the dynamics of the fractional-order system, and the synchronization between pair of fractional order chaotic systems assume considerable significance in the study of nonlinear dynamics. This investigation should also be considered in the near future.
